# The effect of ertugliflozin in patients with nonalcoholic fatty liver disease associated with type 2 diabetes mellitus: A randomized controlled trial

**DOI:** 10.1097/MD.0000000000040356

**Published:** 2024-11-08

**Authors:** Adil Khaliq, Haroon Badshah, Yasar Shah, Inayat Ur Rehman, Kashif Ullah Khan, Long Chiau Ming, Maong Hui Cheng

**Affiliations:** a Department of Pharmacy, Abdul Wali Khan University Mardan, Mardan, Pakistan; b Department of Clinical Pharmacy and Pharmacy Practice, Faculty of Pharmacy, Universiti Malaya, Malaysia; c Department of Medical Sciences, School of Medical and Life Sciences, Sunway University, Bandar Sunway, Malaysia.

**Keywords:** ertugliflozin, nonalcoholic fatty liver disease, type 2 diabetes mellitus

## Abstract

**Background:**

Nonalcoholic fatty liver disease (NAFLD) is a chronic liver disease associated with liver inflammation, fibrosis, and cirrhosis and is associated with a greater risk of hepatocarcinoma. Nonalcoholic steatohepatitis (NASH) is a persistent and progressive form of NAFLD. Recent evidence suggested that ertugliflozin, a sodium-glucose cotransporter 2 inhibitor (SGLT2), suppresses NAFLD development in patients with type 2 diabetes mellitus (T2DM). The objective of this study was to determine the impact of ertugliflozin on improving NAFLD in patients with T2DM and the function of liver enzymes.

**Methods:**

This prospective, randomized, double-blind, placebo-controlled, interventional study aimed to determine the effectiveness of 15 mg of ertugliflozin versus 30 mg of the standard therapy pioglitazone versus placebo in NAFLD patients with T2DM. The study was established based on patient randomization in three groups: ertugliflozin, pioglitazone, and a placebo. This study was registered under the Australian New Zealand Clinical Trial Registry (Trial ID: ACTRN12624000032550).

**Results:**

The impact of therapy was determined in the treatment groups by utilizing liver ultrasonography and biochemical parameters. After 24 weeks of clinical study, the results revealed significant improvement in the grades of fatty liver, especially in the ertugliflozin group. The number of patients with hepatic steatosis significantly decreased among the respective groups classified according to fatty liver grade. Among patients in the ertugliflozin and pioglitazone groups, 45% to 23.4% and 41.7% to 26.6%, respectively, decreased in the Grade 2 group. The aspartate aminotransferase and alanine aminotransferase levels were significantly lower in all the study groups, especially in the ertugliflozin group (*P* ≤ .001).

**Conclusion:**

The present study revealed that the concomitant use of ertugliflozin has favorable effects on liver enzymes, as it decreases liver fat intake and reduces complications in patients with NAFLD-associated T2DM. However, more in-depth studies will be required to observe every aspect of ertugliflozin.

## 1. Introduction

The most common cause of chronic liver disease throughout the world is nonalcoholic fatty liver disease (NAFLD), which is also characterized by liver fibrosis and inflammation and leads to cirrhosis with progression to hepatic carcinoma in some cases.^[1]^ NAFLD is a chronic disorder of dysmetabolic syndrome.^[2,3]^ Most cases of NAFLD are characterized by imbalanced glucose levels, obesity, hypertension, hyperlipidemia, and hepatic expression disorders.^[4]^ This disease affects more than half of the world’s population with obesity and diabetes.^[5]^ The conditions included low-density and high-density lipoprotein, total cholesterol, and hypertriglyceridemia. It has been reported that NAFLD is a presumed cause of chronic liver inflammation in Asian and Western countries.^[6]^ The prevalence rate of NAFLD is 5% to 30%. However, in the world population, a global survey revealed that Pakistan and India are the countries with the most prevalent type 2 diabetes mellitus (T2DM) and obesity.^[6,7]^ Furthermore, NAFLD incidence is very high among individuals with T2DM, and NAFLD incidence is expected to increase further. T2DM and NAFLD are frequently encountered in general practice. Given the complexity of their management, a multidisciplinary approach is often required, incorporating the expertise of gastroenterologists, diabetologists, and internists. This integrated care model is essential for addressing the multifaceted nature of these conditions.^[8]^ T2DM and NAFLD are both highly prevalent in primary care, with T2DM affecting over 422 million people globally^[9]^ and NAFLD affecting 25% to 30% of the general population.^[9,10]^ The conditions are closely linked, especially through insulin resistance, with up to 70% of T2DM patients also having NAFLD.^[10]^ This coexistence significantly increases the risk of cardiovascular disease and liver complications.^[11]^ Primary care physicians play a critical role in the early detection and management of both conditions, which requires a multidisciplinary approach involving specialists in diabetology, gastroenterology, and internal medicine.^[12]^ Effective management focuses on lifestyle changes, pharmacological treatments, and regular monitoring to prevent the progression of these chronic diseases.^[13]^ Nonalcoholic steatohepatitis (NASH) is a destructive disorder of NAFLD characterized by inflammation, hepatic necrosis, and late fibrosis and may further progress to a crucial form of cirrhosis.^[14,15]^ The early transience rate among NASH patients involves hepatocellular carcinoma and liver cirrhosis, along with extrahepatic manifestations, mostly cardiac disease.^[3,16,17]^ The pathogenesis of NAFLD involves multiple factors, including an excessively imbalanced diet and fat-rich food ingestion, along with impaired insulin sensitivity and oxidative stress-induced damage.^[18]^ However, persistent cellular stress and insulin insensitivity may lead to hepatic steatosis, which causes NAFLD and progression to NASH.^[19]^ Recent evidence suggested that a sodium-glucose cotransporter 2 (SGLT2) inhibitor improved NAFLD in patients with T2DM.^[20]^ Ertugliflozin is the newest SGLT2 inhibitor used in patients for the treatment of T2DM. It accelerates urinary glucose excretion, causing a decrease in blood glucose levels, osmotic urination and loss of calories.^[20,21]^ Ertugliflozin improves glucose imbalance and beta cell function, and its effect on insulin sensitivity also decreases body mass.^[22]^ A decrease in insulin insensitivity caused by canagliflozin treatment is very effective in all patients suffering from NAFLD.^[23,24]^ Recently, established studies have shown that SGLT2 inhibitors considerably decrease body weight and body mass index in human trials.^[20,21,25]^ Dysmetabolic iron overload syndrome (DIOS) has been observed in approximately 23% to 38% of patients with NAFLD.^[26,27]^ Hepatic iron accumulation, along with hyperferritinemia, has become a major risk factor in NAFLD patients.^[28]^ Iron plays a vital role in the destruction of the liver and is also a cause of increased insulin insensitivity.^[28]^ Iron accumulates in the body and becomes very toxic to the liver, while lowering iron in the body can overcome such conditions.^[27]^ Moreover, hyperferritinemia in association with NAFLD accelerates hepatocyte injury.^[29]^

There are a few similarities among treatments for T2DM and NAFLD, including alcohol discontinuation, weight reduction, metformin intake, antioxidant use, liraglutide treatment and thiazolidinedione treatment for both conditions.^[30]^ There are no FDA-approved drugs for NAFLD to date.^[14,31]^ Pioglitazone and liraglutide are considerable agents that have been shown to have significant effects on NAFLD associated with T2DM.^[32]^ Ertugliflozin is the most effective and safe oral antidiabetic agent.^[33]^ SGLT2 inhibitors, initially developed for managing hyperglycemia in patients with T2D, have garnered significant interest across various medical fields due to their potential benefits. Studies suggest these agents may improve cardiovascular outcomes, enhance renal function, and offer promising benefits in managing NAFLD, thus broadening their therapeutic scope.^[34–36]^ SGLT2 inhibitors, originally developed for glucose control in T2DM, have demonstrated significant therapeutic benefits beyond diabetes management. They offer cardiovascular protection, as seen in studies showing reduced risk of heart failure and major adverse cardiovascular events,^[24,37]^ and provide renal protection by slowing the progression of chronic kidney disease (CKD), even in non-diabetic patients^[34,35]^ along with SGLT2 inhibitors also reducing serum uric acid level among the gout patients.^[38]^ Additionally, early evidence suggests they may help reduce liver fat in patients with NAFLD, offering potential in managing metabolic liver diseases.^[39]^ These wide-ranging benefits have expanded their use across multiple medical fields, making SGLT2 inhibitors a valuable tool in managing cardiovascular, renal, and metabolic conditions.^[24,40,41]^ Several other SGLT2 inhibitors have been shown to significantly reduce blood sugar, glycosuria, and obesity and improve insulin sensitivity in individuals with T2DM and to produce encouraging results in individuals with NAFLD.^[42,43]^ The aim and objective of this study were to evaluate the effect of ertugliflozin on NAFLD patients with T2DM and its impact on the function of liver enzymes.

## 2. Materials and methods

**Figure 1.**
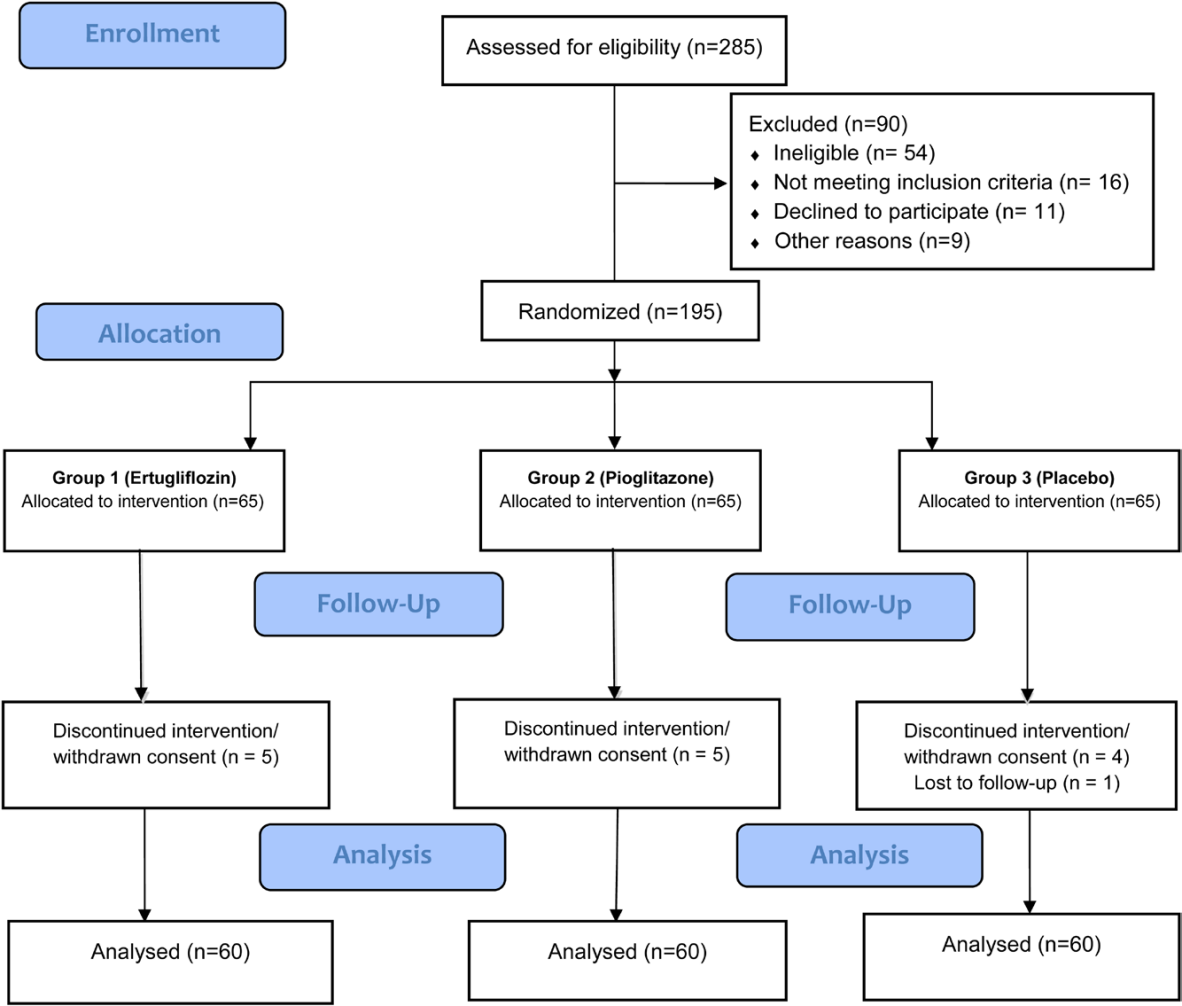
This figure illustrates the flow of participants through different stages of the trial. Out of 285 patients assessed, 90 were excluded due to ineligibility, not meeting criteria, declining participation, or other reasons. The remaining 195 were randomized into three groups of 65: Group 1 (Ertugliflozin), Group 2 (Pioglitazone), and Group 3 (Placebo). Some participants dropped out during follow-up: 5 from Group 1, 5 from Group 2, and 4 from Group 3, with one in Group 3 lost to follow-up. Ultimately, 60 participants from each group were analyzed.

This study is a prospective, randomized, double-blind and placebo-controlled interventional study that was carried out with the intention of determining the effectiveness of ertugliflozin 15 mg oral tablet versus pioglitazone 30 mg versus placebo in NAFLD patients with T2DM.^[44]^ The study was managed in a tertiary care center in Khyber Pakhtunkhwa, Pakistan. The study flow chart is shown in Figure 1.

### 2.1. Study duration and eligibility criteria of participants

This study included adult patients, including males and females aged between 20 and 70 years, with NAFLD and T2DM. The inclusion criterion for participants was fatty liver from grade 1 to grade 3, which was diagnosed via abdominal ultrasonography, along with T2DM.^[45]^ All the patients had elevated liver enzymes associated with NAFLD. The research outcome was obtained within 24 weeks of treatment. The exclusion criteria included patients who had no fatty liver or who had an elevated liver profile caused by other risk factors, such as hepatitis, pregnancy or lactation, alcoholic or alcohol-induced fatty liver disease, biliary illness, smoking cessation, cardiovascular complications, chronic liver ailment, thyroid diseases, cancer, hypolipidemia, renal failure, hypoglycemia, hepatitis, peptic ulcers or the use of herbal medicine and vitamins.^[44]^ Individuals who used any type of medication that can modify or manipulate the function of the liver or who had any drug hypersensitivity were excluded from the study. Laboratory and clinical investigations were performed to confirm the exclusion criteria. Anti-hepatitis B and C antibody tests were performed for each participant to diagnose viral infection.^[24]^

### 2.2. Data collection and participant randomization

The patients’ demographic data and all anthropometric data were collected from the patients’ records. All patients with fatty liver were recruited for the study, and baseline data were collected. A computerized-based randomization sequence technique was followed.^[46]^ The patient was assigned a code for identification. The patients were divided into 3 groups: an intervention group, a control group and a placebo group. Patients were randomized between 3 groups using a 1:1:1 allocation system.^[46,47]^ The study was double-blinded, and the medications were packed into 3 different packages to mask their identities.^[48]^ In the intervention group, each participant received an oral tablet of ertugliflozin 15 mg once a day. In the control group, each patient received 30 mg pioglitazone once a day, while the third group received a placebo daily. All the baseline parameters of the patients were determined at 0 months, follow-up was performed at 3 months (12 weeks), and the final biomarker measurements were recorded at 6 months (24 weeks). The patients were asked about side effects or other symptoms after taking medication.^[46]^

### 2.3. Sample size

A total of 285 participants met the eligibility criteria, and 195 participants who met the eligibility criteria were randomly assigned to receive medications. The sample size of our study was estimated to be 60 participants in each group using protocols used in previous studies.^[47]^ The sample size was decreased due to the withdrawal of consent and discontinuation of treatment. After the randomization of all patients, 180 patients completed the trial (ertugliflozin, n = 60; pioglitazone, n = 60; placebo, n = 60). Informed consent was obtained from each patient.

### 2.4. Ethical considerations and analysis of the data

Written informed consent was acquired from each patient before registering for the study. This study was endorsed by the Ethical and Research Committee of Abdul Wali Khan University (Ethical approval number: EC/AWKUM/2023/18). The study included human subjects and was conducted according to the principles and guidance of the Declaration of Helsinki.^[49]^ This study was also registered under Australian New Zealand Clinical Trail Registry (Trial ID: ACTRN12624000032550).

Statistical analysis was performed using SPSS 27 (Chicago). The descriptive statistics were applied to the demographics of all patients and are presented as percentages with means and standard deviations among all variables. The measurements were taken at different times for each group. The analysis is based on the intention to treat (ITT) among all the patients who received the medication.^[44]^ The analysis was performed within the groups through paired sample t-tests, while within groups, one-way ANOVA and the chi-squared test were used. A *P* value of less than < .001 was considered to indicate statistical significance.^[46,50]^

### 2.5. Anthropometric indices

Height and body weight were measured by an automatic scale and a stadiometer attached to the wall. Body mass index (BMI) was measured by using the formula weight in kilograms (kg)/height in meters (m).^[44]^ All the measurements were taken in an upright position without shoe soles, as shown in Table 1.

**Table 1:** Comparison of demographic characteristics of the patients between the groups

Parameters	Pioglitazone	Ertugliflozin	Placebo	Total	*P* value
Gender					
Male	50 (83.4%)	50 (83.4%)	47 (78.4%)	157 (87.2%)	.136†
Female	10 (16.6%)	10 (16.6%)	13 (21.6%)	23 (12.8%)	
weight in kg (mean ± SD)	94.53 ± 8.72	92.16 ± 5.75	90.53 ± 5.37	92.41 ± 6.94	.173‡

† Chi-square test.

‡ Maan–Whitney *U* test.

### 2.6. Biochemical parameters

The body weight and BMI of each participant were measured throughout the study. Biochemical parameters such as aspartate aminotransferase (AST), alanine aminotransferase (ALT), alkaline phosphatase (ALP), and gamma-glutamyl transferase (γ-GGT) were measured occasionally. The normal ranges of ALT were 7 to 41 U/L, AST was 12 to 38 U/L, γ-GGT was 9 to 58 U/L, and ALP was 33 to 96 U/L.^[5,26]^ Higher ALT or AST levels, i.e., ALT > 42 U/L and AST > 39 U/L, were considered abnormal liver enzymes.^[51]^ Low-density lipoprotein cholesterol (LDL-C), high-density lipoprotein cholesterol (HDL-C), triglyceride (TG), and blood sugar (BS) levels were analyzed enzymatically on a Microlab 300 autobiochemical analyzer using Diasys kits (Holzheim, Germany). All laboratory equipment was calibrated adequately as per standard standards. Glycated hemoglobin (HbA1c), uric acid, the fibrosis-4 (FIB-4) index, and ferritin were monitored initially and then after 3 and 6 months.^[52]^ The FIB-4 index and homeostatic model assessment for insulin resistance (HOMA-IR) were identified by their formula equations.^[53,54]^

### 2.7. Liver ultrasonography

Liver imaging was performed through a Mindray DP-10 diagnostic ultrasound machine with a convex transducer at a frequency of 3.5 to 5.0 MHz. The ultrasonic diagnosis of fatty liver was performed primarily during enrollment, in the 12th week and after the 24th week. Diagnostic imaging was performed on the same ultrasound system and by the same radiologist to prevent errors in patient assessment.^[44]^ Fat content in the liver was determined by ultrasound on the basis of sonographic echogenicity, which classified fatty liver into various grades (0–3). Sonologists describe fatty liver as grade 0 (liver with no fat accumulation), grade 1 (presence of low fat causing a mild increase in echogenicity), grade 2 (moderate agglomeration of fat with slight loss of visual echogenicity of the branch wall of portal veins without disappearance of the diaphragm) or grade 3 (high-grade echogenicity with severe visual absence of echogenicity of portal vein branches and with diaphragm obstruction).^[46]^ The four quadrants and the surface of the liver were observed and monitored through ultrasound.^[55]^

### 2.8. Outcomes

A reduction in the fat content of liver hepatocytes was the primary outcome of this study. Moreover, the secondary outcomes included improvements in the function of liver enzymes as well as possible improvements in the levels of abnormal biochemical factors, specifically ALT, AST, total cholesterol, TG, HDL-C, LDL-C, ALP, and HOMA-IR 44.

## 3. Results

Among the total patients, 157 (87.2%) were male, and 23 (12.8%) were female; these patients were uniformly distributed among the three groups. The recruitment period for this trail was from August 10, 2023 to August 31, 2023. The average weight of the patients was 92.41 ± 6.94 kg at 0 weeks, as shown in Table 1. All of the patients were distributed into 5 age groups in which the number of participants aged 31 to 40 years increased, followed by those aged 18 to 30 years, as shown in Table 2. The average patient age was 38.0 ± 11.4 years. The body weight of patients decreased markedly in the ertugliflozin group as well as in the pioglitazone group, while the decrease in body weight in the placebo group was not significant.

**Table 2:** Comparison on age-wise distribution of the NAFLD patients between the groups

Age groups	Pioglitazone	Ertugliflozin	Placebo	Total	*P* value
18–30 years	13	14	12	39	<.001*
31–40 years	14	15	14	43	
41–50 years	12	11	10	33	
51–60 years	12	11	14	37	
61 years and above	9	10	9	28	
Age in years (Mean ± SD)	38.28 ± 8.42	39.45 ± 12.63	37.17 ± 11.09		

Chi-square test.

NAFLD = nonalcoholic fatty liver diseases.

* *P* values < .05 indicate statistical significance.

An ultrasound of the liver revealed improvement in NAFLD patients in all three groups (ertugliflozin, pioglitazone, and placebo). The liver fat content in the ertugliflozin and pioglitazone groups was notably reduced. However, the percentage of patients with grade 0 fatty liver increased to 16.6% in the ertugliflozin therapy group and 10% in the pioglitazone therapy group, while after the intervention, the percentage increased to 1.6% in the placebo group, as shown in Table 3. All the patients completed the study.

**Table 3:** Comparison among the grades of fatty liver at initial assessment and after 24 weeks of interventional treatment in NAFLD patients

Pre-treatment				Post-treatment		
	Pioglitazone (n = 60)	Ertugliflozin (n = 60)	Placebo (n = 60)	Pioglitazone (n = 60)	Ertugliflozin (n = 60)	Placebo (n = 60)
Grade 0	0 (0%)	0 (0%)	0 (0%)	6 (10.00%)	10 (16.6%)	1 (1.6%)
Grade 1	16 (26.6%)	16 (26.6%)	17 (28.4%)	26 (43.4%)	32 (53.4%)	15 (25%)
Grade 2	25 (41.7%)	27 (45%)	24 (40%)	16 (26.6%)	14 (23.4%)	24 (40%)
Grade 3	19 (31.7%)	17 (28.4%)	19 (31.6%)	12 (20.0%)	4 (6.6%)	20 (33.4%)

NAFLD = nonalcoholic fatty liver diseases.

The levels of serum ALT significantly decreased from 86.6 (IU/L) to 34.4 (IU/L) after the use of ertugliflozin for 24 weeks (*P* ≤ .001). However, the serum γ-GGT and serum AST levels decreased markedly from 80 (IU/L) to 48.5 (IU/L) and from 98.5 (IU/L) to 45.2 (IU/L), respectively, after treatment with ertugliflozin for 24 weeks (*P* < .001). The serum level of HbA1c decreased from 7.2% to 6.1% (*P* ≤ .001) in the ertugliflozin group, while in the pioglitazone group, it decreased from 7.4% to 6.8% (*P* = .065). The serum TG concentration decreased significantly from 260 (mg/dL) to 195 (mg/dL) after treatment with ertugliflozin for 24 weeks (*P* ≤ .001), as shown in Table 4.

**Table 4:** The biomarker results of Ertugliflozin at initial analysis and after 24 weeks of treatment of patients with NAFLD

Parameters	Baseline	6 months	*P* value
Aspartate aminotransferase (AST) (IU/L)	98.5 ± 25.5	45.2 ± 20.0	<.001*
Alanine aminotransferase (ALT) (IU/L)	86.6 ± 50.4	34.4 ± 23.0	<.001*
Alkaline phosphatase (ALP)	187.0 ± 58.9	170.6 ± 42.2	.77
Gamma-glutamyl transferase (γ-GGT) (IU/L)	80 ± 55.4	48.5 ± 50.1	<.001*
Total Cholesterol	195.5 ± 30.7	185.5 ± 24.3	<.001*
Low-density lipoprotein (LDL) (mg/dL)	130.3 ± 25.4	120.3 ± 21.4	.025
High-density lipoprotein (HDL) (mg/dL)	51.6 ± 11.7	57.1 ± 15.6	.006
Triglycerides (TG) (mg/dL)	260 ± 126.1	195 ± 99.5	.013
Blood sugar (BS) (mg/dL)	192 ± 39.5	142 ± 32.5	<.001*
Glycated hemoglobin (HbA1C) (%)	7.2 ± 1.8	6.1 ± 1.0	.010
HOMA-IR	3.99 ± 0.53	3.10 ± 0.45	.001*
Insulin (μU/mL)	18.9 ± 10.1	11.3 ± 9.4	<.001*
Uric acid (UA) (mg/dL)	7.67 ± 1.44	5.1 ± 1.11	.004
Ferritin (ng/mL)	198.5 ± 169.1	153.4 ± 101.7	.017
Fibrosis-4 (FIB-4 index)	1.82 ± 0.46	1.04 ± 0.43	.006
Body weight (kg)	92.16 ± 5.75	80.5 ± 4.68	<.001*
BMI (kg/m^2^)	31.8 ± 3.8	22.4 ± 3.2	<.001*

Data are shown as the mean ± standard deviation.

NAFLD = nonalcoholic fatty liver diseases.

* *t* paired sample tests were performed within the groups.

Body weight significantly decreased from (92.16 ± 5.75) kg at the initial measurement to (80.5 ± 4.68) kg after 24 weeks of ertugliflozin treatment (*P* < .001). The FIB-4 index ratio decreased from (1.82 ± 0.46) at the initial assessment to (1.04 ± 0.43) after 24 weeks of ertugliflozin treatment. Similarly, the impacts on other biochemical parameters are also presented in Table 4.

A total of 180 patients participated in this research and continued the trial until the end of the study. The BMI of the patients in the ertugliflozin group was significantly lower (22.4 ± 3.2) than that in the pioglitazone (25.2 ± 3.6) and placebo (31.5 ± 3.9) groups, as shown in Table 5. In the analysis, there was a prominent variation among the ertugliflozin and pioglitazone groups and between the ertugliflozin and placebo groups in terms of body weight. The results of the present study showed that differences in the body weights of the subjects had no marked effect on other biochemical parameters measured in the trial, such as liver enzymes and fatty liver grade. BMI is the most authentic indicator for NAFLD assessment, but it also has no impact on other variables.

**Table 5:** Biochemical parameters of NAFLD patients at initial assessment and after the 24 weeks of intervention

Parameters	Pioglitazone (n = 60)			Ertugliflozin (n = 60)			Placebo (n = 60)			*P* value†
	Baseline	24 weeks	*P* value	Baseline	24 weeks	*P* value	Baseline	24 weeks	*P* value	
BS (mg/dL)	202.5 ± 30.5	158.4 ± 27.5	<.001*	192 ± 39.5	142 ± 32.5	<.001*	190.2 ± 35.4	215.4 ± 33.4	.18	.017
HbA1C (%)	7.4 ± 1.6	6.8 ± 1.5	.075	7.2 ± 1.8	6.1 ± 1.0	.010	7.0 ± 2.00	8.0 ± 2.6	.48	.007
Insulin (μU/mL)	14.5 ± 8.6	10.8 ± 3.7	.046	18.9 ± 10.1	11.3 ± 9.4	<.001*	17.92 ± 9.1	16.90 ± 9.0	.53	.014
TG (mg/dL)	245.4 ± 133.2	200 ± 89.7	.005	260 ± 96.1	195 ± 80.5	.003	267.1 ± 78.8	270.2 ± 76.2	.57	.009
Chol (mg/dL)	194.5 ± 27.2	188.1 ± 26.4	<.001*	195.5 ± 30.7	185.5 ± 24.3	<.001*	182 ± 32.7	185.4 ± 33.5	.20	.002
LDL (mg/dL)	128.6 ± 22.2	122.7 ± 19.5	.35	130.3 ± 25.4	120.3 ± 21.4	.025	122.5 ± 24.6	124.7 ± 22.8	.85	.256
HDL (mg/dL)	44.0 ± 16.4	47 ± 18.3	.021	51.6 ± 11.7	57.1 ± 15.6	.006	48.9 ± 10.8	50.4 ± 12.0	.46	.023
ALT (U/L)	96 ± 17.7	75.8 ± 14.6	.03	86.6 ± 50.4	34.4 ± 23.0	<.001*	95 ± 12.5	100.2 ± 12.5	.30	.571
AST (U/L)	90 ± 18.4	40.5 ± 17.7	.01	98.5 ± 25.5	45.2 ± 20.0	<.001*	95.5 ± 20.4	99.5 ± 19.8	.12	.081
ALP (U/L)	185.7 ± 58.8	170.3 ± 40.3	<.001*	187.0 ± 58.9	170.6 ± 42.2	.77	176.1 ± 30.5	174.5 ± 32.2	.07	.671
γ-GGT (IU/L)	89.6 ± 54.3	51.4 ± 37.5	.035	80 ± 55.4	48.5 ± 50.1	<.001*	85.7 ± 45.9	86.5 ± 46.6	.34	.025
Fibrosis-4 Index	1.79 ± 0.51	1.2 ± 0.36	.003	1.82 ± 0.46	1.04 ± 0.43	.006	1.75 ± 0.44	1.85 ± 0.40	.09	.064
HOMA-IR	3.60 ± 0.59	3.30 ± 0.62	.019	3.99 ± 0.53	3.10 ± 0.45	.001*	3.70 ± 0.55	3.75 ± 0.54	.56	.066
BMI (kg/m^2^)	30.7 ± 4.8	25.2 ± 3.6	.004	31.8 ± 3.8	22.4 ± 3.2	<.001*	30.1 ± 3.8	31.5 ± 3.9	.49	.001

ALP = alkaline phosphatase, ALT = alanine aminotransferase, AST = aspartate aminotransferase, BMI = body mass index, BS = blood sugar, HDL-C = high-density lipoprotein cholesterol, HOMA-IR = homeostatic model assessment for insulin resistance, LDL-C = low density lipoprotein cholesterol, NAFLD = nonalcoholic fatty liver diseases, TG = triglyceride, γ-GGT = gamma-glutamyl transferase.

* A *t* paired sample test was performed within the groups.

† *P* value for the difference between the groups.

Our study revealed that AST, ALT, and ALP levels were decreased in the drug-treated groups after the interventions. On the other hand, the levels of AST, ALT, and ALP remained unchanged in the placebo group after 6 months of treatment. A comparison of the differences between the ALT and AST levels within the experimental groups revealed that the liver enzymes decreased more in the ertugliflozin group, while the ALP level decreased significantly in the pioglitazone group, as shown in Table 5. Our results showed that ertugliflozin was significantly effective in both experimental groups. The study revealed that ertugliflozin was effective at lowering TG levels from (260 ± 96.1 mg/dL) to (195 ± 80.5 mg/dL) (*P* = .003), ALT levels from (86.6 ± 50.4 U/dL) to (34.4 ± 23.0 U/dL) (*P* < .001) and insulin resistance HOMA-IR levels from (3.99 ± 0.53) to (3.10 ± 0.45) (*P* = .001). The effects of ertugliflozin on various parameters were adequately compared to those of the control (pioglitazone) and placebo, as shown in Table 5.

The plasma levels of TG, LDL, and Chol decreased significantly in the ertugliflozin group and pioglitazone group (control), while no decrease was observed in the placebo group after 24 weeks of intervention. However, the decrease in the ertugliflozin group was significant (*P* ≤ .05). Insulin sensitivity increased significantly (*P* < .001) in the ertugliflozin group. BS and HbA1c also decreased among the groups, as predicted. BMI was an important parameter and had a vital impact on this study. The decrease in BMI among the drug-treated groups was effective. In our study, the ertugliflozin group had a significantly lower BMI (31.8 ± 3.8 to 22.4 ± 3.2 kg/m^2^) (*P* ≤ .001) than the pioglitazone group (30.7 ± 4.8 to 25.2 ± 3.6 kg/m^2^) (*P* = .007). One-way ANOVA showed a significant difference between the groups (*P* = .001^**+**^) in terms of BMI.

## 4. Discussion

The focus of the present study was to determine the outcome of ertugliflozin (an SGLT2 inhibitor) in NAFLD patients with T2DM. The results of the study showed that treatment with ertugliflozin improved the incidence of Grade 3 fatty liver disease to Grade 2 and Grade 1 fatty liver disease, while the morbidity ratio decreased significantly. The results also showed that ertugliflozin improved the serum ALT, AST, and γ-GGT levels and significantly reduced the weight of patients with NAFLD and T2DM.

Few research studies have shown that plasma HbA1c is 0.5% to 0.7% lower in patients treated with ertugliflozin than in those treated with a placebo.^[56,57]^ Our results revealed that the plasma levels of HbA1c decreased by 1.0% in the case of ertugliflozin and 0.6% in the case of pioglitazone, while the placebo had no significant effect. However, the current changes were greater than those in previous studies.^[33,56]^ The reasons behind such outcomes are the use of higher doses of ertugliflozin for longer periods of time, a proper diet and regular exercise.

Totade et al^[58]^ reported that the administration of ertugliflozin regulates blood glucose and prevents hypoglycemia in T2DM patients. Lingvay et al^[57]^ reported that ertugliflozin was effective in hyperglycemic patients with uncontrollable blood sugar. Another study reported that ertugliflozin had marked effects on controlling high fasting blood glucose levels in patients with a long history of T2DM.^[59–61]^ In the respective study, the blood glucose level decreased within 24 weeks of therapy with ertugliflozin. The results of our study suggested that lowering blood glucose was a significant outcome of ertugliflozin administration. However, pioglitazone also significantly decreased blood sugar. This decrease was lower than that reported in a previous trial in patients with diabetes mellitus.^[56,62]^ The current protocol improves random plasma glucose levels and insulin resistance.

Recent studies have shown that the body weight of patients receiving SGLT2 inhibitors for 13 months is approximately 3.3 kg less than that of patients receiving a placebo.^[56]^ Regardless, SGLT2 inhibitors accelerate glucose secretion from the body along with other body fluids. Glucose is excreted through renal diuresis. A study reported that the excretion of glucose from the body negatively affects energy consumption, which may lead to a reduction in body weight.^[24,33,46,56]^ Similarly, our findings demonstrated that the body weight of the study participants significantly decreased upon ertugliflozin treatment, and it continuously decreased until the end of the study (24 weeks). This study suggested that in-depth investigation of the phenomenon of greater reductions in body weight resulting from ertugliflozin than from other SGLT2 inhibitors will be mandatory.

There are no such data available regarding TG reduction with ertugliflozin intervention. However, a study reported that NAFLD patients treated with canagliflozin had significantly reduced serum TG levels.^[55]^ Similarly, in our study, the TG ratio was also lower in patients treated with ertugliflozin than in those in the placebo and control (pioglitazone) groups. Serum HDL and LDL throughout the study remained the same in the case of placebo, although ertugliflozin showed a potential effect. The mechanism associated with a reduction in the TG ratio of ertugliflozin treatment remains unclear. However, a study needs to be performed regarding the effect of ertugliflozin on lipid metabolism. A decrease in the serum TG concentration in response to ertugliflozin was reported for the first time in this study.

Iwata et al^[38]^ reported that dapagliflozin notably lowered uric acid in NAFLD patients. A study performed by Ferreira et al^[35]^ revealed that empagliflozin has an impact on serum uric acid levels via metabolism. Our study revealed that the serum uric acid concentration was notably decreased in the ertugliflozin treatment group. These findings revealed that ertugliflozin decreased uric acid levels. It has also been reported in a study that empagliflozin aids uric acid removal by facilitating glucose transporter-9.^[41]^ Most studies have suggested that serum uric acid levels have a potential effect on kidney function and cause liver toxicity in patients with hepatic steatosis.^[35,41]^

Our study also revealed that ertugliflozin improved liver dysfunction and hepatic steatosis in NAFLD-associated patients with T2DM. The key factor of study accomplishment involved the treatment of liver dysfunction and the significant loss of body weight after 24 weeks of ertugliflozin administration. The improvement in insulin resistance as well as glycemic control continued in patients with NAFLD and T2DM. In NASH and NAFLD patients, the loss of body weight is an important element for improving liver function deterioration and other parameters. There are no specific data available for the effect of ertugliflozin on NAFLD or NASH. However, few studies have measured the effect of SGLT2 inhibitors in NAFLD patients with T2DM.^[24]^

Furthermore, lowering body weight improves insulin levels, insulin resistance, and other aspects of a healthy life.^[16,31]^ Our study revealed that body weight and insulin levels coexist and further decrease as body weight decreases over a duration of 24 weeks. Rametta et al^[27]^ reported that ferritin levels decreased as ALT decreased in patients treated with canagliflozin. Similarly, in the present study, the ferritin and ALT levels decreased further over 24 weeks. However, no therapeutic outcomes were observed, but a parallel decrease was noted in this study. The excess load of iron in the liver can cause insulin resistance due to metabolic dysfunction, which can modify the release of adiponectin.^[28,63]^ It is also responsible for the development of other metabolic diseases, such as type 1 and T2DM and obesity.^[63]^ The onset of NAFLD in patients is characterized by insulin resistance, oxidative stress, hyperferritinemia, metabolic disorders and T2DM.^[22,61]^

Previous studies have shown that canagliflozin, an SGLT2 inhibitor, has a significant impact on reducing the FIB-4 index.^[30]^ On the other hand, dapagliflozin, which belongs to the same class, has no prominent effect on the FIB-4 index.^[48,64]^ We used ultrasonography to assess the grade of fatty liver and the fibrosis-4 index to analyze the risk of liver fibrosis. The FIB-4 index decreased markedly after ertugliflozin treatment. Ertugliflozin commits to decreasing FIB-4 index values and serum ferritin levels, along with the subduing of liver fibrosis and the progression of hepatocellular carcinoma. This improvement in FIB-4 was first reported for ertugliflozin treatment.

Our study revealed that ertugliflozin treatment causes a decrease in HbA1c, AST, BS, ALT, TG, γ-GTP, uric acid, body weight, ferritin, and the FIB-4 ratio among NAFLD patients after 24 weeks. The ertugliflozin administration is also responsible for an increase in glucose excretion through diuresis, which decreases glucose levels in the blood, decreases body weight, and improves insulin insensitivity. Furthermore, abnormal lipid metabolism and impaired glucose tolerance also occur.

SGLT2 inhibitors have emerged as a key therapeutic option for managing a range of interconnected metabolic disorders, including diabetes, cardiovascular disease, CKD, and NAFLD. The intricate nature of these conditions necessitates a synergistic, multidisciplinary approach, involving the coordinated expertise of gastroenterologists, diabetologists, cardiologists, and nephrologists. This collaboration ensures a holistic, evidence-based care model that addresses the interrelated physiological systems, optimizing patient outcomes.^[24,65]^ SGLT2 inhibitors have demonstrated significant benefits across these areas by improving glycemic control, reducing the risk of cardiovascular events, and protecting renal function.^[66]^ Furthermore, these inhibitors show promise in managing NAFLD by reducing liver fat and improving liver-related outcomes, particularly in patients with concurrent diabetes and cardiovascular disease.^[67]^ The coordinated involvement of various healthcare specialists is essential to ensure that treatment plans address the full spectrum of a patient’s health needs, thus optimizing therapeutic outcomes and enhancing quality of life.^[65,68]^ This holistic strategy has proven effective in preventing disease progression and improving long-term health in patients with multiple metabolic conditions.^[66,67]^

Recent research has significantly expanded the therapeutic scope of SGLT2 inhibitors beyond their established use in diabetes management. Emerging evidence suggests that these agents offer substantial benefits in cardiovascular protection, independent of their glucose-lowering effects.^[36]^ Clinical trials have demonstrated reduced risks of heart failure hospitalizations and cardiovascular death in both diabetic and non-diabetic populations.^[24,69]^ Moreover, SGLT2 inhibitors have been shown to slow the progression of CKD by reducing intraglomerular pressure, proteinuria, and renal inflammation, thus enhancing overall renal function.^[70]^ This nephroprotective effect is particularly beneficial in patients with diabetic nephropathy, but recent studies indicate similar outcomes in individuals with CKD unrelated to diabetes.^[24,37,71]^ Furthermore, growing interest surrounds the role of SGLT2 inhibitors in managing NAFLD. These agents are thought to improve hepatic insulin sensitivity and reduce liver fat content, potentially lowering the progression to steatohepatitis and cirrhosis.^[39,40]^ Collectively, these findings underscore the expanding therapeutic potential of SGLT2 inhibitors in managing cardiovascular, renal, and hepatic comorbidities, positioning them as versatile agents in clinical practice.

Research has extensively explored non-pharmacological measures such as diet and physical activity in improving NAFLD. The Mediterranean diet coupled with regular physical activity has shown substantial improvements in liver health, particularly when patients adhere to lifestyle changes over time.^[72,73]^ Studies highlight that lifestyle interventions, including dietary strategies and exercise, have the potential to significantly improve or even reverse NAFLD.^[72,74]^ Furthermore, multidisciplinary approaches that integrate diet, physical activity, and behavioral therapy have proven effective in enhancing liver function and reducing hepatic fat content.^[32,67,75]^ Another key finding emphasizes the combination of nutritional adjustments with physical activity, which leads to improved metabolic markers and a decrease in liver fat.^[76,77]^ Notably, increasing adherence to the Mediterranean diet also improves oxidative stress and inflammation, both of which play a critical role in NAFLD progression.^[78,79]^ Additionally, few studies suggested the probiotics supplementation showed a potential response in improving NAFLD.^[80,81]^ These studies collectively underline the effectiveness of non-pharmacological approaches, demonstrating that structured diet and lifestyle changes can lead to significant improvements in liver health and overall metabolic function.

Limitations of our study includes: The study was performed on small number of patients, future multicentered studies while recruiting large sample of patients needed to be performed. Furthermore, no information on diet taken by patients and whether any non-pharmacological intervention performed by patients were not collected and we realized it after performing analysis. Future studies should consider these aspects and this is considered as one of the possible limitations of our study.

## 5. Conclusion

This randomized, double-blind, placebo-controlled study provides strong evidence that ertugliflozin significantly improves liver function and reduces fatty liver severity in patients with NAFLD associated with T2DM. Over the course of 24 weeks, ertugliflozin demonstrated a marked reduction in liver fat grades and a notable improvement in liver enzyme profiles (ALT, AST, γ-GGT), alongside improvements in metabolic parameters, such as triglycerides and body weight. These findings align with ertugliflozin’s established benefits in glucose management and extend its potential therapeutic role into the treatment of NAFLD, a common and serious comorbidity in patients with T2DM.

Given the current lack of FDA-approved treatments for NAFLD, this study underscores the promise of SGLT2 inhibitors as a novel therapeutic approach, particularly for patients with comorbid metabolic conditions. However, while the study demonstrated significant short-term benefits, long-term studies are needed to further explore the sustained impact of ertugliflozin on liver health and its potential role in preventing the progression of NAFLD to more severe liver conditions, such as cirrhosis or hepatocellular carcinoma.

In summary, ertugliflozin represents a promising treatment option for managing both glycemic control and liver health in NAFLD patients with T2DM. Future research should focus on extended treatment durations, varying dosages, and its broader impact on cardiovascular and renal outcomes to fully establish its place in clinical practice.

## Acknowledgments

The authors would like to extend their sincere gratitude to all the patients, investigators, and clinical researchers who contributed to this study. Special thanks are owed to Dr Sifat Ullah, an Endocrinologist, whose dedication and efforts were instrumental in making this study possible.

## Authors consent

We confirm that the manuscript has been read and approved by all named authors and that there are no other persons who satisfied the criteria for authorship but are not listed. We further confirm that the order of authors listed in the manuscript has been approved by all of us.

We confirm that we have given due consideration to the protection of intellectual property associated with this work and that there are no impediments to publication, including the timing of publication, concerning intellectual property. In so doing we confirm that we have followed the regulations of our institutions concerning intellectual property.

We understand that the Corresponding Author is the sole contact for the Editorial process (including the Editorial Manager and direct communications with the office). He is responsible for communicating with the other authors about progress, submissions of revisions, and final approval of proofs. We confirm that we have provided a current, correct email address which is accessible by the Corresponding Author.

## Author contributions

**Conceptualization:** Adil Khaliq, Haroon Badshah, Yasar Shah.

**Data curation:** Adil Khaliq.

**Formal analysis:** Adil Khaliq, Haroon Badshah.

**Funding acquisition:** Adil Khaliq.

**Investigation:** Adil Khaliq, Kashif Ullah Khan, Long Chiau Ming, Maong Hui Cheng.

**Methodology:** Adil Khaliq, Kashif Ullah Khan, Long Chiau Ming, Maong Hui Cheng.

**Project administration:** Adil Khaliq, Long Chiau Ming.

**Resources:** Adil Khaliq, Haroon Badshah, Yasar Shah, Inayat Ur Rehman.

**Software:** Inayat Ur Rehman.

**Supervision:** Haroon Badshah, Yasar Shah, Inayat Ur Rehman.

**Validation:** Haroon Badshah, Yasar Shah, Inayat Ur Rehman.

**Visualization:** Adil Khaliq, Yasar Shah, Inayat Ur Rehman, Maong Hui Cheng.

**Writing – original draft:** Adil Khaliq, Kashif Ullah Khan.

**Writing – review & editing:** Adil Khaliq, Long Chiau Ming, Maong Hui Cheng.
